# Hypoxia-activated genes from early placenta are elevated in Preeclampsia, but not in Intra-Uterine Growth Retardation

**DOI:** 10.1186/1471-2164-6-111

**Published:** 2005-08-29

**Authors:** Daniel Vaiman, Françoise Mondon, Alexandra Garcès-Duran, Thérèse-Marie Mignot, Brigitte Robert, Régis Rebourcet, Hélène Jammes, Sonia T Chelbi, Frédérique Quetin, Geoffrey Marceau, Vincent Sapin, François Piumi, Jean-Louis Danan, Virginie Rigourd, Bruno Carbonne, Françoise Ferré

**Affiliations:** 1 Génétique et Epigénétique des Pathologies Placentaires, GEPP, U709 INSERM-Université René Descartes-Institut Alfred Jost, Pavillon Baudelocque, Hôpital Cochin, 123 Boulevard de Port-Royal, 75014, Paris, France; 2 Laboratoire de Biochimie Medicale, Faculte de Medecine et de Pharmacie, UMR INSERM U.384 UA, 28 Place Henri Dunant, BP. 38, 63000 Clermont-Ferrand, France; 3 Centre de Ressources Biologiques, Laboratoire de Radiobiologie et d'Etudes des Génomes, Centre de Recherches INRA de Jouy-en-Josas, INRA, CRJJ, 78352 Jouy-en-Josas, France; 4 UPR CNRS 9078, Université René Descartes ParisV, Site Necker, 156 rue de Vaugirard, 75015 Paris, France; 5 Service de Réanimation Néonatale, Institut de Puériculture et de Périnatalogie, 26, boulevard Brune, 75014 Paris, France; 6 Service de Gynecologie-Obstetrique, Hopital Saint Antoine, 184 rue du Faubourg Saint Antoine, 75012 Paris, France; 7 Département de Génétique Animale, INRA, 78352, Jouy-en-Josas,, France

## Abstract

**Background:**

As a first step to explore the possible relationships existing between the effects of low oxygen pressure in the first trimester placenta and placental pathologies developing from mid-gestation, two subtracted libraries totaling 2304 cDNA clones were constructed. For achieving this, two reciprocal suppressive/subtractive hybridization procedures (SSH) were applied to early (11 weeks) human placental villi after incubation either in normoxic or in hypoxic conditions. The clones from both libraries (1440 hypoxia-specific and 864 normoxia-specific) were spotted on nylon macroarrays. Complex cDNAs probes prepared from placental villi (either from early pregnancy, after hypoxic or normoxic culture conditions, or near term for controls or pathological placentas) were hybridized to the membranes.

**Results:**

Three hundred and fifty nine clones presenting a hybridization signal above the background were sequenced and shown to correspond to 276 different genes. Nine of these genes are mitochondrial, while 267 are nuclear. Specific expression profiles characteristic of preeclampsia (PE) could be identified, as well as profiles specific of intra-uterine growth retardation (IUGR).

Focusing on the chromosomal distribution of the fraction of genes that responded in at least one hybridization experiment, we could observe a highly significant chromosomal clustering of 54 genes into 8 chromosomal regions, four of which containing imprinted genes. Comparative mapping data indicate that these imprinted clusters are maintained in synteny in mice, and apparently in cattle and pigs, suggesting that the maintenance of such syntenies is requested for achieving a normal placental physiology in eutherian mammals.

**Conclusion:**

We could demonstrate that genes induced in PE were also genes highly expressed under hypoxic conditions (P = 5.10^-5^), which was not the case for isolated IUGR. Highly expressed placental genes may be in syntenies conserved interspecifically, suggesting that the maintenance of such clusters is requested for achieving a normal placental physiology in eutherian mammals.

## Background

Preeclampsia (PE) is a severe disorder of human pregnancies affecting up to 10 % of primiparous women in industrialized countries. This hypertensive disease develops from the second half of pregnancy and is associated with proteinuria, and sometimes with oedemas. The causes of preeclampsia are complex and multiple, with a combination of environmental and genetic effects from maternal [1] as well as paternal origin [2,3]. At the histological level, preeclampsia is characterized by a shallow colonization of the maternal endometrium (and more specifically of the wall of the uterine vessels) by the invasive cytotrophoblasts. In a normal gestation, this process occurs during the second trophoblastic colonization wave around the end of the first trimester of gestation allowing the invasive cytotrophoblasts to reach the placental bed. In this case, the arterial wall is infiltrated by endovascular trophoblasts, triggering a suppression of the vasomotor control, thus resulting in a very important dilatation of the lumen and a loss of the elastic properties of the arteries [4-8]. By contrast, when the invasion is defective, remaining too shallow [9], fibrinous material accumulates in the arteries, myocytes proliferate. This may lead to local thrombosis and is therefore supposed to impact on the local oxygen pressure leading to placental ischemia/hypoxia, and ultimately to functional anomalies of the maternal vascular endothelium [10,11]. Finally, the functions of the syncytiotrophoblast, the specific tissue resulting from the fusion of the cytotrophoblasts, may be modified, leading to trophoblast apoptosis. Links between placental pathology and hypoxia are now clearly documented ([12,13], for a recent review, see Challier et Uzan, 2003; [14]). Intra-Uterine Growth Retardation (IUGR) constitutes another group of complex diseases, a large subset of which is associated with placental malfunction, and often with preeclampsia, although this is not at all systematic. IUGR may be defined as a rupture in the normal growth curve of the foetus, although it is generally defined as a birth weight inferior to the 10^th ^or to the 3^rd ^percentile (according to a chosen arbitrary limit) of the smallest babies. This simple definition does not take into account the dynamics of the growth curve, but is very easily workable. This explains at least partly the underlying complexity present under the term IUGR. At least, two very distinct situations are possible: IUGR may be the result of endogenous developmental and growth factors affecting the fetus growth; or, alternatively, a placental defect may inhibit the transfer of nutriments and oxygen from the mother to the fetus (IUGR of vascular origin or not). In the following, vascular IUGR correspond to those exhibiting an abnormal Doppler. In this latter case, the IUGR may be caused by a vascular pathology such as PE. Consequently, there is a clear need for classification of IUGR, and for improving the understanding of its etiology, both issues possibly based upon the characterization of specific marker genes.

In addition, it is now well documented that babies presenting with a small birth weight are at increased risk for developing systemic pathologies at adulthood, such as diabetes or cardiovascular troubles [15,16]. One clear limitation of the therapeutic possibilities for PE and IUGR resides in the fact that whilst many indications suggest that their causes are very precocious during placental development [17], their symptoms occur late, at least from mid-gestation, and more often from the last trimester of the pregnancy. Evaluating the risk early would allow orienting the medical choices towards a better follow-up of the pregnancy, or even towards some pharmacological options, such as the chronic use of mild doses of aspirin.

**Figure 1:**
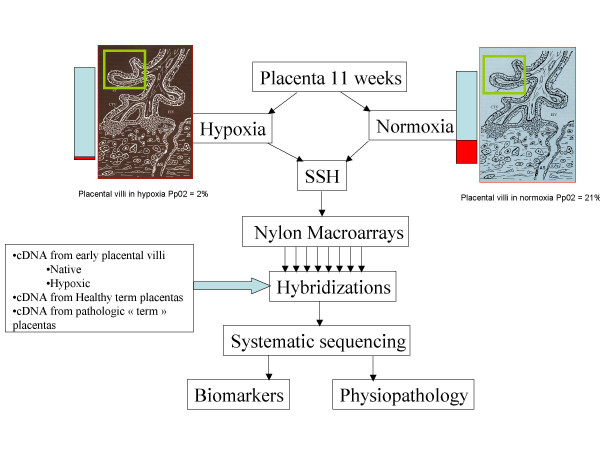
A chart presenting the protocol used to construct the principal tool used in this study: high density nylon membranes spotted with two Suppressive Subtractive Hybridization libraries (SSH). The original cDNAs were obtained from 11-weeks placental villi maintained in normoxia or hypoxia. Two reciprocally subtracted libraries were constructed and spotted at high density on nylon membranes. Then hybridizations were carried out using complex probes from various placentas (either from healthy, or from pathological pregnancies). The rationale of using early villi and hybridizing with near-term villi was the aim of identifying genes modified early by hypoxia, and still modified later chronically in the pathological state.

In order to explore on a wide basis the links between hypoxia and placental diseases, and to identify early putative markers of the preeclamptic pathology, we combined the Subtractive/Suppressive Hybridization (SSH) methodology starting with cDNA material obtained from first trimester placental purified villi with the construction of high-density nylon macroarrays (Figure 1). A first characterization of the libraries has previously been performed by systematic sequencing, enabling to identify all the genes that were modulated by short-term hypoxia in early placental villi, even when this genes are expressed at a low level [18]. However, this systematic sequencing approach does not indicate which gene could constitute an optimal early marker for disease detection (highly and differentially expressed). Therefore, in the present study, we used the less sensitive approach of hybridization in order to identify genes modulated early by hypoxia in pregnancy (the end of first trimester, which is crucial for trophoblast invasion), and also highly expressed and modified in placental diseases occurring later. We report here on the macroarray hybridization results obtained from 20 different sources of cDNA. We demonstrate a clear association between PE and the early hypoxic induction of a series of genes involved in different metabolic processes. This association was totally absent when cDNAs from isolated IUGR were used as probes. We could also demonstrate that the kinetics of hypoxia is not linear, and that genes transcriptionnally modulated in early placentas after 3 h of hypoxia may return to their basal expression level when the culture is maintained for 48 h in hypoxic conditions. Finally, we demonstrate that genes highly expressed in the placenta are clustered in specific chromosome regions, in particular in regions previously defined as containing imprinted genes, such as 11p15.5.

## Results

### 1. Overview of the genes highly expressed in placental physiology according to their putative interactions

**Figure 2:**
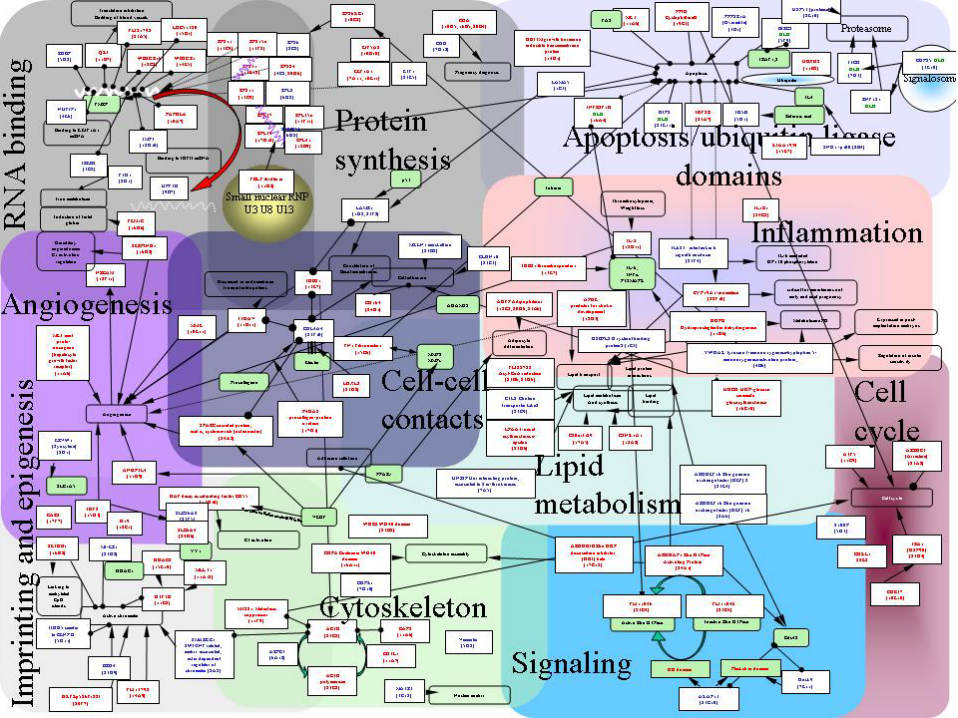
Putative physiological relationships between nuclear genes found expressed at a detectable level on the membranes. Amongst a total of 269 nuclear genes, known described relationships could be deduced from the literature for 117 of them. The genes were grouped in 11 categories. In blue and red are represented genes transcriptionnally inhibited or activated by hypoxia, respectively. In green are presented genes that were not detected by hybridization but that may play critical roles in placental physiology. Open boxes present the main physiological action of several of these genes. Arrows indicate an activation effect, while lines terminated by circles indicates an inhibitory effect. Lines terminated by two circles are indicative of a physical interaction between two protein products, or between a protein and a RNA molecule. Table 5 (supplemental table) gives the complete name of the genes displayed on the figure.

When all the hybridizations were considered, 641 clones out of 2304 yielded a response above the background level of the membrane after an overnight autoradiography. Hybridization with a labeled Oligo dT indicated that 281 clones corresponded to Oligo dT sequences cloned during the SSH procedure. These clones were generally highly labeled in most hybridization experiments, except when the probe was prepared from a placenta where the apparent level of transcriptional activity was dramatically decreased (i.e. vascular IUGR, see below). Finally, 360 sequences corresponded to known genes of which 276 were different (9 mitochondrial and 267 nuclear genes). The normalization effect of the SSH was demonstrated by the fact that 243 genes were found only once. The most frequent was *CGA *(corresponding to the common alpha chain of four glycoprotein hormones, LH, FSH, TSH and hCG), found in 9 occurrences. The other frequently represented genes corresponded to mitochondrial genes involved in the structure of the mitochondrial ribosome (*16S*, 6 occurrences, and *12S*, 4 occurrences) or in the respiratory chain (*COX1*, 4 occurrences, *COX3*, 3 occurrences). The complete set of genes could be divided in 11 distinct cell functions: RNA binding, Protein synthesis, Apoptosis, Inflammation, Cell to Cell contacts, Angiogenesis, Epigenetic mechanisms and Imprinting, Cytoskeleton constitution, Signal transduction, Cell cycle and Lipid metabolism. These functions and the links existing between 117 genes are represented in Figure 2, drawn from literature information. Genes induced by hypoxia are written in red and genes inhibited by hypoxia in blue. Amongst several noticeable features, the picture exhibits a large amount of genes encoding RNA-interacting factors. Several of these genes encode proteins recognizing specific mRNAs. This is the case for NUFIP1 that interacts specifically with *EEF1A1 *mRNA, this latter encoding a specific elongation factor interacting with ribosomal proteins for elongating nascent polypetidic chain. Similarly, IGFII (Insulin-like Growth Factor II) mRNA Binding Protein-3 (IMP-3), interacts in particular with IGFII mRNA [19], a well known very important actor of placental growth and physiology, as demonstrated recently by the specific invalidation of the IGFII placental isoform [20]. The importance of IMP3 was recently emphasized in a study demonstrating by RNA interference its involvement for enhancing IGFII mRNA translation in K562 leukemia cells [21]. RNA interacting factors represent less than 1% of the total gene content in mammals. In our subset of highly expressed placental genes, they represent over 4% of the total.

### 2. Hierarchical clustering of genes and hybridization experiments

**Figure 3:**
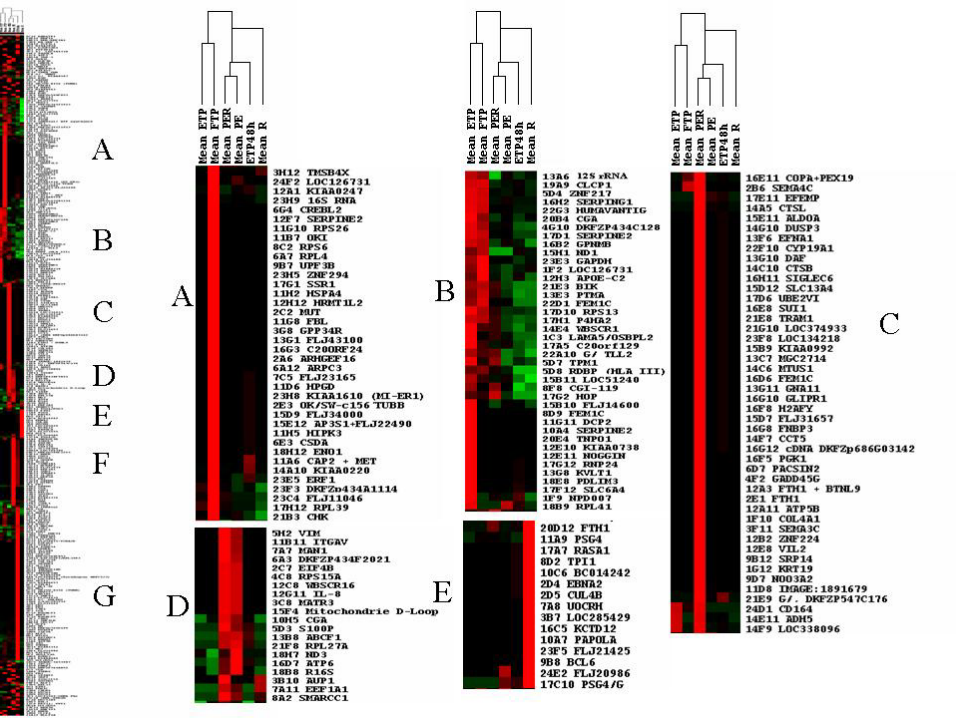
Pictures obtained after data clustering of the SSH hybridizations using the Treeview software [22]. The programs were used according to the parameters described in Material and Methods. Clusters of genes expressed in specific situations are represented. Above the general tree are presented the means of the different hybidization grouped into 6 categories of probes used. Clusters of transcriptionnally induced genes could be characterized. A, Full Term Placentas (Mean FTP); B, Early Term Placentas (Mean ETP), C, Preeclampsia with IUGR (mean PER); D, PER + isolated PE; E, isolated IUGR (Mean R), F, isolated PE (Mean PE), G, 48 h hypoxia.

**Figure 4:**
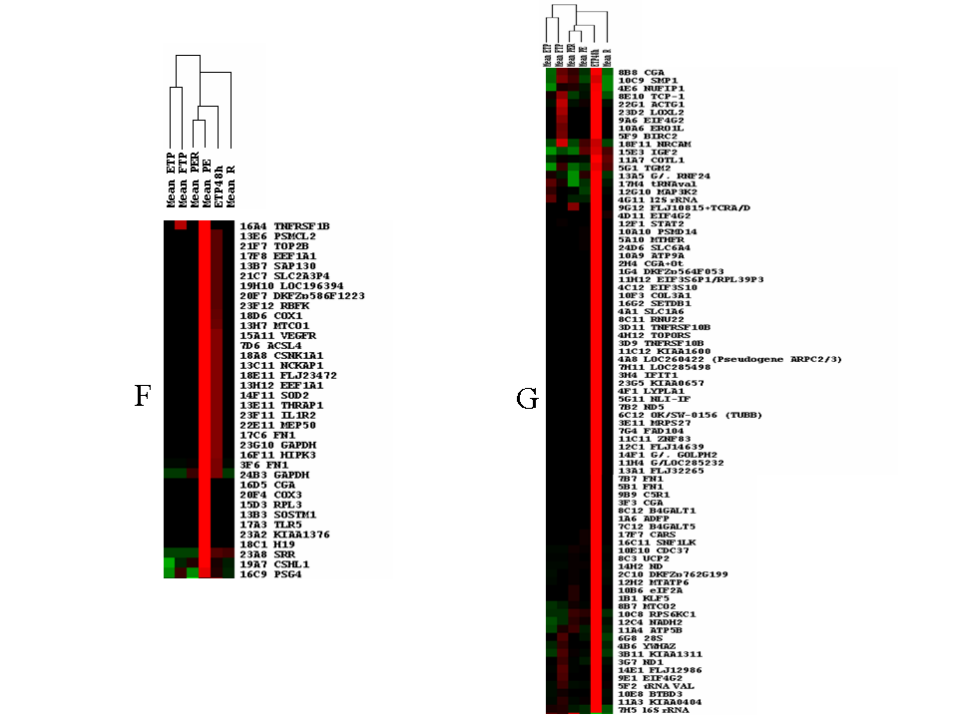
Pictures obtained after data clustering of the SSH hybridizations using the Treeview software [22]. The programs were used according to the parameters described in Material and Methods. Clusters of genes expressed in specific situations are represented. Above the general tree are presented the means of the different hybidization grouped into 6 categories of probes used. Clusters of transcriptionnally induced genes could be characterized. A, Full Term Placentas (Mean FTP); B, Early Term Placentas (Mean ETP), C, Preeclampsia with IUGR (mean PER); D, PER + isolated PE; E, isolated IUGR (Mean R), F, isolated PE (Mean PE), G, 48 h hypoxia.

**Figure 5:**
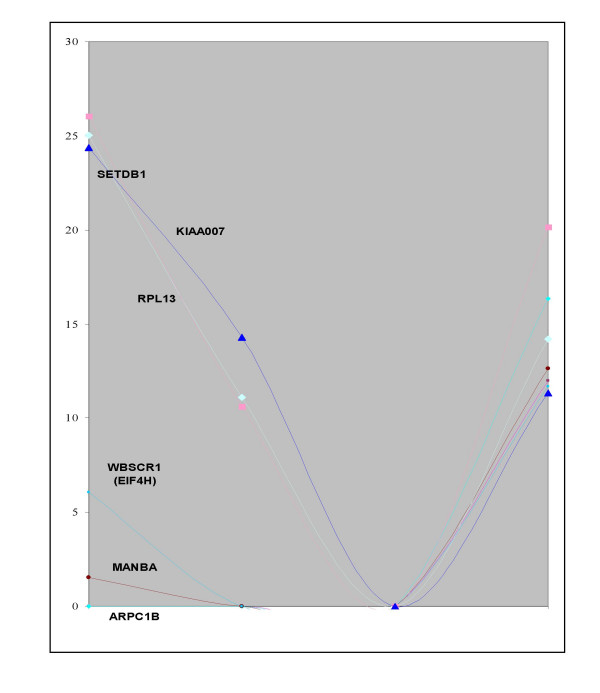
Kinetics of hypoxia regulation in early (11 weeks) placentas. Six examples of genes exhibiting a transcriptional arrest under short hypoxic conditions, but coming back to almost normal levels of expression under extended hypoxic conditions.

To take into account the complexity of the tissue, the hybridization experiments were grouped into six categories from the cDNA prepared from samples listed in Table 1. The signals from the different experiments were averaged, as described in Experimental Procedures. The clustering tool developed by Eisen and coworkers [22] was applied to the set of 360 identified genes. Two hundred and seventy five genes could be grouped in seven clusters of similar expression (A to G, figure 3 and 4). The experiments were classified by the program in the following organization: normal placenta, either term or early were grouped into one cluster while placenta from disease states or from villi maintained in hypoxia during 48 h were grouped in another cluster. In a lower order cluster, PE and PER were grouped and associated with early villi maintained during 48 h in hypoxia while isolated IUGR was placed separately. The distribution of genes into two sets (hypoxia-induced versus hypoxia-inhibited) made it possible to assess statistically the possible relationships between the effects on the transcript levels of a relatively short period of hypoxia (3 h) on early placenta (the hypoxic condition that was used to construct the arrays) and pathological states developing later (Table 2). Genes that are specific of the placenta, either early or at term, have a slight trend to be specifically induced by hypoxia in early term placenta. There is also a highly significant tendency of preeclamptic villi, either from isolated PE or PE combined with IUGR, to express genes induced by 3 h hypoxia in early placental villi. The inverted trend is observed at a highly significant level in hybridizations carried out with probes obtained from early term villi maintained in hypoxia during 48 h with an excess of "normoxic" genes found in this situation. We supposed that this observation could be related to a specific kinetics of induction by oxygen concentration, where short exposure to hypoxia may have effects that are at the opposite of long-term hypoxia. Indeed, we could observe that genes whose expression was modified by short periods of hypoxia may later return to their basal expression level as shown by analyzing kinetics of expression in hybridizations with cDNA of the same placenta maintained in hypoxia during 3, 24 or 48 hours (Figure 5). In the cases of isolated vascular IUGR, due to a drastic limitation of the materno-foetal blood flow only 40 positive clones yielded a detectable signal (instead of 400–500 when other probes were hybridized to the membranes, including the polyA containing clones). These positive clones corresponded to mitochondrial genes, *IGFII *and *PSG4*, *PSG5*, and *PSG7 *(Pregnancy Specific Glycoproteins 4, 5 and 7), indicating that these genes constitute a minimal survival set to sustain gestation. Moreover, the signal level of positive clones was quite comparable with that of other experiments, demonstrating that the relevant RNA species are indeed present at a high level. Since transcriptional activity is very reduced in vascular IUGR (as shown by the very low abundance of polyA+ RNAs), this suggests that some mRNA molecules are specifically protected from degradation in the very harsh pathological condition of vascular IUGR.

**Table 1:** Status of the patients used in the study

**Patient**	**Status**	RT (*)	**Weeks Amenhorrhea**	**Year mother**	HTA (**)	**Proteinuria g/24 h**	Uterine Doppler (****)	**Oligoamnios**	**Apgar 1**	**Apgar 5**	**SEX**	**Birthweight in g (Percentile)**
3008	Term (control)	784	38+4	32	normal	normal	normal	normal	10	10	M	3050
7497	Term (control)	816	38+0	34	normal	normal	normal	normal	10	10	M	3270
497	Term (control)	817	40+5	35	normal	normal	normal	normal	10	10	M	3040
3007	Term (control)	818	38+4	43	normal	normal	normal	normal	10	10	M	3430
3011	Term (control)	819	38+0	37	normal	normal	normal	normal	10	10	M	3710
3013	Term (control)	820	39+4	42	normal	normal	normal	normal	10	10	F	2740
3017	Term (control)	821	38+5	41	normal	normal	normal	normal	10	10	F	3110
												
3004	PE	765	28+5	34	205/120	10,69	normal	normal	2	8	M	830
3005	PE	766	37+4	45	140/90	0,17	Unilateral	normal	10	10	F	2930
225	PE	807	27+0	24	145/90	2,7	normal	normal	9	10	M	1080
242	PE	867	33+1	26	150/95	5,36	normal	normal	8	10	M	1960
3010	PE	808	32+5	36	160/110	14,36	normal	normal	10	10	M	1196
50	PE	885	30+0	22	175/105	8,68	normal	normal	8	10	M	1390
												
4003	PE+IUGR	915	34+1	36	180/120	2,1	normal	amniotic fluid in excess	0	7	F	1670 (< 5)
3012	PE+IUGR	770	34+1	37	140/90	6,98	normal	unknown	9	10	F	1800 (between 5 and 10)
3016	PE+IUGR	772	28+0	37	160/100	nc	bilateral	Abnormal	7	10	M	780 (< 5)
												
3003	vascular IUGR	773	37+3	26	160/90	normal	bilateral	unknown	10	10	M	1890 (between 5 and 10)
3021	vascular IUGR	810	31+3	35	normal	normal	bilateral	Abnormal	7	10	M	1380 (> 10 but ***)
3022	vascular IUGR	814	37+0	33	normal	normal	unknown	Abnormal	3	9	M	2330 (between 5 and 10)

* RT: reversed transcribed RNA sample

** HTA: Arterial Hypertension

*** IUGR defined bya break in the intrauterine growth curve, a bilateral doppler associated with an absence of diastolic pressure

**** Presence of a doppler signal in one or both the umbilical arteries

**Table 2:** Statistics of gene induction under various conditions

**Category**			**Normoxia (plates 1–9)**	**Hypoxia (plates 10–24)**	**Chi2**	**Observations**
A	Full-Term Placenta	Observed	12	26		
		Expected	14,25	23,75	0,451	38
B	Early+Full-Term Placenta	Observed	9	31		
		Expected	15	25	**0,050**	40
C	PE+RCIU	Observed	9	37		
		Expected	17,25	28,75	**0,012**	46
D	PE+RCIU and isolated PE	Observed	10	10		
		Expected	7,5	12,5	0,248	20
E	RCIU	Observed	6	9		
		Expected	5,625	9,375	0,841	15
F	Isolated PE	Observed	2	35		
		Expected	13,875	23,125	**0,000**	37
G	Early Term Placenta Hypoxia 48 h	Observed	45	36		
		Expected	30,375	50,625	**0,001**	81

Each category (A-G) corresponds to a cluster of genes observed in figure 3 (a-b). The expected values were calculated from the proportion of clones in each subtracted library. Significant Chi2 values are represented in bold characters

### 3. Identification of factors specific of the pathological status of the placenta

Table 3 was extracted from the database using PE as a keyword in the field "Maximal signal". It contains 56 different genes, amongst a total of 71. Among these genes, some were observed in only one PE case (27), while some others were observed in several or all the hybridizations with probes obtained from PE (29 signals), 6 in PE +IUGR, 6 only in the case of a severe PE that was used in the hybridization (clinically defined as an arterial hypertension exceeding 160 mm Hg), while the 3 left could also be observed at a high level in normal term placentas. The set of genes highly expressed in more than one PE constitutes of course a collection of natural candidates for a further exploration of the pathology. These 17 genes are: *ACTG1*, *ATP5B*, *ATP6*, *ANGPTL4*, *CGA*, *COX1*, *COX3*, *CSHL1 *(human Placental lactogen), *GAPDH*, *FLJ22728*, *H19*, *ND1*, *ND3*, *NUFIP1*, *PSG5*, *PSG7 *and *RPL41*. Some of these genes, such as *CSHL1 *have already been identified as PE markers [23,24]. Some others such as *ANGPTL4*, *COX1*, *FLJ22728*, *H19 *and *NUFIP1 *are completely new candidates. There were 22 signals corresponding to genes highly elevated in IUGR corresponding to 20 different factors, 15 of which were not correlated with PE (Table 4).

**Table 3:** Genes induced in PE

Gene symbol	Gene name	Maximal signal	Protein category	Chromosomal localization	Library address
16S ribosomal RNA	16S ribosomal RNA	one PE case	Transcription/Translation	Mitochondrie	18B8
16S ribosomal RNA	16S ribosomal RNA	PE+IUGR	Mitochondrial metabolism	Mitochondrie	14H2
18S rRNA	ARN 18S	severe PE	Transcription/Translation		13F12
ACTG1	actin, gamma 1	several PE	Structure protein	17q25	22G1
angiopoietin-like 4	ANGPTL4 = PPARG angiopoietin related protein	several PE	Transcription/Translation	19p13.3	11D9
ATP5B	ATP synthase, H+ transporting, mitochondrial F1 complex, beta polypeptide	several PE	Mitochondrial metabolism	12p13-qter	11A4
ATP6	ATP synthase F0 subunit 6	several PE	Mitochondrial metabolism	Mitochondrie	16D7
C9orf90	chromosome 9 open reading frame 90 DKFZp762G199	one PE case		9q34.13	2C10
CDC37	CDC37 cell division cycle 37 homolog (S. cerevisiae)	several PE	Cell cycle	19p13.2	10E10
CGA	glycoprotein hormones, alpha polypeptide	several PE	Signal transduction	6q12-21	23B10
CGA	glycoprotein hormones, alpha polypeptide	several PE	Signal transduction	6q12-21	2C5
CGA	glycoprotein hormones, alpha polypeptide	several PE	Signal transduction	6q12-21	23D12
CGA	glycoprotein hormones, alpha polypeptide	several PE	Signal transduction	6q12-21	10H5
CGA	glycoprotein hormones, alpha polypeptide	several PE	Signal transduction	6q12-21	2H4
CGA	glycoprotein hormones, alpha polypeptide	several PE	Signal transduction	6q12-21	16D5
CGA	glycoprotein hormones, alpha polypeptide	Terms/PE	Signal transduction	6q12-21	8B8
COX1	Cytochrome c oxidase subunit I	several PE	Mitochondrial metabolism	Mitochondrie	13H7
COX1	Cytochrome c oxidase subunit I	several PE	Mitochondrial metabolism	Mitochondrie	15B3
COX1	Cytochrome c oxidase subunit I	several PE	Mitochondrial metabolism	Mitochondrie	12H9
COX1	Cytochrome c oxidase subunit I	several PE	Mitochondrial metabolism	Mitochondrie	18D6
COX2	Cytochrome c oxidase subunit II	one PE case	Mitochondrial metabolism	Mitochondrie	8B7
COX3	Cytochrome c oxidase subunit III	several PE	Mitochondrial metabolism	Mitochondrie	20F4
CSHL1	chorionic somatomammotropin hormone-like 1	several PE	Signal transduction	17q24.2	19A7
CSNK1A1	Casein kinase 1, alpha 1	one PE case	Signal transduction	5q32	18A8
DDX3X	DEAD (Asp-Glu-Ala-Asp) box polypeptide 3, X-linked	one PE case	Transcription/Translation/Modifi	p11.3-11.23	17F11
DKFZP434F2021	DKFZP434F2021 protein	one PE case		3q13.2	6A3
DKFZp586F1223	Hs.28540	severe PE		11q23	20F7
EEF1A1	eukaryotic translation elongation factor 1 alpha 1	one PE case	Transcription/Translation	6q14.1	7A11
EIF4B	eukaryotic translation initiation factor 4B	one PE case	Transcription/Translation	12q13.13	2C7
ERVWE1	endogenous retroviral family W, env(C7), member 1 (syncytin)	one PE case	Cell-cell contacts	7q21-22	8G1
FEM1C	fem-1 homolog c (C.elegans)	PE+IUGR	Transcription/Translation	5q22	16D6
FEM1C	fem-1 homolog c (C.elegans)	PE+IUGR	Transcription/Translation	5q22	8D9
FLJ11149	riboflavin kinase	one PE case	Transport	9q21.31	23F12
FLJ22728	hypothetical protein FLJ22728 DKFZp761I1913	several PE	Transport	11p15.2	23G6
FLJ22728	hypothetical protein FLJ22728 DKFZp761I1913	several PE	Transport	11p15.2	23B6
GAPD	glyceraldehyde-3-phosphate dehydrogenase	several PE	Mitochondrial metabolism	12p13	23D3
GAPD	glyceraldehyde-3-phosphate dehydrogenase	several PE	Mitochondrial metabolism	12p13	24B3
GLIPR1	GLI pathogenesis-related 1 (glioma)	PE+IUGR		12q21.1	16G10
H19	H19, imprinted maternally expressed untranslated mRNA	several PE	RNA gene	11p15.5	18C1
H3F3B	H3 histone, family 3B (H3.3B)	severe PE	Chromatin structure	17q25	11C8
IL8	interleukin 8	severe PE	Apoptose regulation	4q13-q21	12G11
ITGAV	integrin, alpha V (vitronectin receptor, alpha polypeptide, antigen CD51)	one PE case	Cell-cell contacts	2q31-32	11B11
LAMA5	laminin, alpha 5	one PE case	Apoptose regulation	20q13.2-13.3	1C3
LOC126731	LOC126731	Terms/PE		1q42.13	24F2
LOC374933	Homo sapiens LOC374933 (LOC374933),	PE+IUGR		1p36.33	21G10
MAN1	integral inner nuclear membrane protein	one PE case	Transcription/Translation/Modifi	12q14	7A7
MATR3	Matrin 3	severe PE	Transcription/Translation/Modifi	5q31.3	3C8
MGC2714	hypothetical protein MGC2714	PE+IUGR		11q22.2	13C7
ND1	NADH dehydrogenase subunit 1	several PE	Mitochondrial metabolism	Mitochondrie	12C4
ND3	NADH dehydrogenase subunit 3	several PE	Mitochondrial metabolism	Mitochondrie	18H7
NRCAM	neuronal cell adhesion molecule	one PE case	Cell-cell contacts	7q31.1-q31.2	18F11
NUFIP1	nuclear fragile × mental retardation protein interacting protein 1	several PE	RNA-interacting factor	13q14	4E6
OSBPL2	oxysterol binding protein-like 2	one PE case	Signal transduction	20q13.3	1C3
PSG4	pregnancy specific beta-1-glycoprotein 4	one PE case	Signal transduction	19q13.2	16C9
PSG4	pregnancy specific beta-1-glycoprotein 4	one PE case	Signal transduction	19q13.2	11A9
PSG5	pregnancy specific beta-1-glycoprotein 5	several PE	Signal transduction	19q13.2	10F12
PSG7	pregnancy specific beta-1-glycoprotein 7	several PE	Signal transduction	19q13.2	10C11
PSG7	pregnancy specific beta-1-glycoprotein 7	several PE	Signal transduction	19q13.2	16F9
RPL41	ribosomal protein L41	several PE	Transcription/Translation/Modifi	12q13	18B9
RPS11	ribosomal protein S11	one PE case	Transcription/Translation/Modifi	19q13.3	13C9
RPS24	ribosomal protein S24	one PE case	Transcription/Translation/Modifi	10q22-23	4C8
RPS6KC1	ribosomal protein S6 kinase, 52kDa, polypeptide 1	severe PE	Transcription/Translation/Modifi	1q41	10C8
rRNA 28S	Human 28S ribosomal RNA gene	one PE case	RNA gene	8q21.1-q21.2	6G8
S100P	S100 calcium binding protein P	one PE case	Cell cycle	4p16	5D3
SMARCC1	SWI/SNF related, matrix associated, actin dependent regulator of chromatin, subfamily c, member 1	one PE case	Structure de la chromatine	3p23-21	8A2
SRP9	signal recognition particle 9kDa	one PE case	Transport	1q42.13	5E10
UCP2	uncoupling protein 2 (mitochondrial, proton carrier)	one PE case	Mitochondrial metabolism	11q13	8C3
VIM	vimentin	one PE case	Structural protein	10p13	5H2
WBSCR1	Williams-Beuren syndrome chromosome region 1 EIF4H	one PE case	Transcription/Translation/Modifi	7q11.23	14E4
WBSCR16	Williams-Beuren syndrome chromosome region 16	one PE case	Transcription/Translation/Modifi	7q11.23	12C8
YWHAZ	tyrosine 3-monooxygenase/tryptophan 5-monooxygenase activation protein, zeta polypeptide	Terms/PE	Signal transduction	8q23.1	4B6

**Table 4:** Genes induced in IUGR

Gene symbol	Gene name	Maximal signal	Protein category	Chromosomal localization	Library address
16S ribosomal RNA	16S ribosomal RNA	PE+IUGR	Mitochondrial metabolism	Mitochondria	14H2
BC014242	Hs.5064	IUGR		5	10C6
cDNA DKFZp686G03142	Homo sapiens mRNA; cDNA DKFZp686G03142	IUGR		5	16G12
COTL1	coactosin-like 1 (Dictyostelium)	IUGR	Structural protein	16q24.1	11A7
CUL4B	cullin 4B	IUGR	Cell cycle	Xq23	2D5
DAF	decay accelerating factor for complement (CD55, Cromer blood group system)	IUGR	Signal transduction	1q32	13G10
FEM1C	fem-1 homolog c (C.elegans)	IUGR+PE	Transcription/Translation	5q22	8D9
FEM1C	fem-1 homolog c (C.elegans)	IUGR+PE	Transcription/Translation	5q22	16D6
FTH1	ferritin, heavy polypeptide 1	IUGR	Transport	11q13	20D12
FTH1	ferritin, heavy polypeptide 1	IUGR	Transport	11q13	2G1
GLIPR1	GLI pathogenesis-related 1 (glioma)	IUGR+PE		12q21.1	16G10
IMAGE:3453987	Homo sapiens cDNA clone IMAGE:3453987	IUGR		4	10C5
IGF2	Insulin-like growth factor 2 (somatomedin A)	IUGR	Growth factor	11p15.5	15E3
IMP-3	IGF-II mRNA-binding protein 3	IUGR	Transcription/Translation/Modification	7p11	18G10
KIAA1354	KIAA1354 protein	IUGR	Signal transduction	9p22	2D4
LOC285429	hypothetical protein LOC285429	IUGR		4p14	3B7
LOC374933	Homo sapiens LOC374933 (LOC374933),	PE+IUGR		1p36.33	21G10
MGC2714	hypothetical protein MGC2714	PE+IUGR		11q22.2	13C7
ND1	NADH dehydrogenase subunit 2	IUGR	Mitochondrial metabolism	Mitochondria	15H1
PAPOLA	poly(A) polymerase alpha	IUGR	Transcription/Translation/Modification	14q32.31	10A7
SND1	staphylococcal nuclease domain containing 1 EBNA2 coactivator p100	IUGR	Transcription/Translation	7q31.3	2D4
TPI1	triosephosphate isomerase 1	IUGR	Transport	12p13	8D2

### 4. Promoter structure of PE/Hypoxia induced genes

Sequences spanning from 5000 bp of DNA 5' of the transcription start site and extending 200 bp after, were recovered from GenBank at the NCBI and were analysed for their composition in CpG islands and the presence or absence of Hypoxia Inducible Factor 1 α (HIF1α) binding sites. HIF is a transcription factor protected from degradation in many cell system under hypoxic conditions. It plays an essential role in modulating responses to hypoxia by inducing or inhibiting multiple genes. Four possible binding sites have been described for this factor, ACGTGC, ACGTGG, GCGTGC and GCGTGG [25].

The promoters could be classified into four categories: Several of them, such as the promoters of *PSG4*, *5 *or *7*, *CUL4*, or *CGA*, do not contain any noticeable CpG islands. Several others contain a concentration of CpG islands very close to, or encompassing the transcription start site (*PAPOLA*, *WBSCR1*, *COTL1*, *SMARCC1*). Other promoters contain CpG islands at around 2000 bp 5' upstream of the ATG (*FEM1C*, *FLJ11149*). Finally, some promoters are highly enriched in CpG islands over the whole 5000 bp examined (*ACTG1*, *IGFII*, *H19*). The set of promoters with the CpG density and the position of putative HIF binding sites are supplied as Supplemental data file 1. We could also classify these promoters according to the maximal density of CpG achieved in the 5 kb window analysed in five groups (less than 50, from 50 to 100, 100 to 150, 150 to 200 and more than 200). Consistently with the high GC-richness of putative HIF binding site, there was a clear linear relationship between the CpG density and the number of putative HIF binding sites (R^2 ^= 0.86). The distribution of these sites was analysed inside the promoters by sharing these promoters into two halves of equal length and counting the putative sites inside each subsequence. A student T-test did not reveal any preference towards one side against the other (P = 0.45).

### 5. Non-random chromosome distribution of placental genes in eutherian mammals

The analysis of the cytogenetic location available from our database revealed that several genes expressed at a detectable level in hybridization experiments were clustered to specific chromosomal regions. We focused our interest on the precise chromosome location of these genes, using the information available at the NCBI site [26] in order to obtain their precise physical position expressed in megabases.

**Figure 6:**
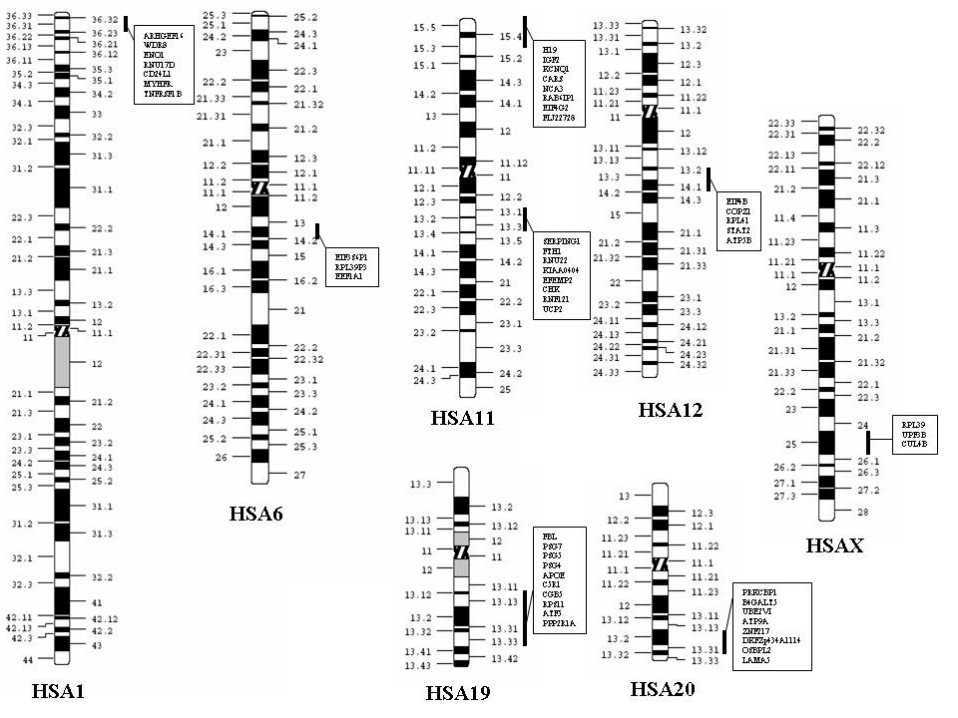
Non-random chromosomal clustering of genes highly expressed in the placenta. Statistical analysis of intergenic distances revealed the existence of 8 clusters distributed on 7 chromosomes. The clusters located on 1q36, 11p15.5, 19q13 and 20q13 are known to belong to imprinted chromosomal regions.

We could confirm by statistical analysis (see Experimental Procedures) that the distribution of the subset of highly expressed placental genes was not random. On the contrary, we identified 8 clusters on 7 different chromosome regions: 1p36 (9.76 Mb), 6q14 (0.23 Mb), 11p15 (11.63 Mb), 11q13 (16.28 Mb), 12q13 (3.65 Mb), 19q13 (12.35 Mb), 20q13 (15.11 Mb) and Xq24 (7.45 Mb) (Figure 6). Among these regions, 4 are known to contain imprinted genes (1p36, 11p15, 19q13 and 20q13). These four regions are conserved in synteny and colinearity in mice, while the regions corresponding to human 11q13 and 12q13 are separated on different mice chromosomes. Despite the more limited mapping information available in pigs, obtained at the Iccare website [27] and cattle [28,29], we did not detect any chromosome breakpoints in these species for these specific chromosome regions.

## Discussion

In this study we designed a new transcriptome resource directed at evaluating the effects of hypoxia on human placenta. This tool is particularly original if compared to the existing commercial membranes, since the distribution of clones into two subgroups makes it possible to analyze statistically whether one condition is connected to hypoxia-induced stimulation or inhibition of gene expression. This was clearly shown for PE, a pathologic condition that correlated very well with the induction of "hypoxic" genes. Since the placenta is one of the organs presenting the greatest abundance of diversified transcripts, these membranes can also be useful for characterizing either other biological systems, or the effects of hypoxia on other tissues. The use of the SSH approach to construct the membranes may also generate some biases as the cloning is dependent on the existence of *Rsa1 *restriction sites in the starting material (driver and tester cDNAs). Nevertheless, our tool, focused on early hypoxia, is a useful complement to other DNA arrays experiments, based on commercial membranes [30]. As shown in our study, this tool was used to analyze at the mRNA level the consequences of the two most frequent placental pathologies, preeclampsia and Intra-Uterine Growth Retardation.

Some of the genes found associated with PE in the present study had already been evaluated as putative markers of placental pathological status, such as human chorionic gonadotrophin (hCG) and human placental lactogen (hPL, also known as CSHL1), as well as pregnancy specific glycoproteins (PSGs). A significant serum increase in hCG was found more prevalent in preeclamptic women [23]. In another recent study, hPL and PSGs were found reduced at 17 weeks in the serum of patients who develop later a preeclamptic condition, albeit serum levels are restored later [31]. hCG is composed of two polypeptidic chains, α and β, encoded by *CGA *(common to four polypeptide hormones) and *CGB*, respectively. It is known to play important roles in placental physiology [32]. In our study *CGA *was indeed found induced in several PE cases, but could not be associated with a specific differential oxygen status. *CGB *was present in the membranes, but was not specifically induced in preeclampsia. The only *CGB *clone was located in plate 7H12 (normoxia). The over-expression of *CGA *could lead to an overall increase of hCG in the patient serum however the mechanisms involved for regulating the expression of the two polypeptidic chains constitutive of hCG seem to display opposite modes of regulation.

Recently, Bersinger and Odegard [31] have demonstrated that in IUGR, hPL is continuously lowered later in pregnancy. This is consistent with our results, as we could not detect any trace of expression of this gene in the IUGR probes that were hybridized on the membranes. Similarly, the expression level was low in control term placentas (FTP). However, hPL appeared strongly induced in PE, especially in severe cases. These results at the mRNA level differ from the findings of Bersinger and Odegard [31] concerning the hPL protein levels, which were going back to normal at weeks 28 and 33 of preeclampsia. Possibly, the accumulation of hPL mRNA would not be followed by translation in PE.

Only three PSG were found in the membranes amongst the 11 existing genes. PSGs are supposed to play an essential immunomodulatory effect in pregnancy [33-35]. Consistently with our result, in a recent microarray study [36] the authors used a commercial membrane to identify differentially expressed genes in preeclamptic and normal placentas. Among the ten most highly expressed genes in the membranes, the authors have identified PSG4, PSG5 and PSG7. Interestingly, those are exactly the highly expressed genes that we have found in term placentas suggesting that only these PSGs are specifically expressed at a high level in term pregnancies. This observation raises the question of the regulation of the entire cluster of PSG genes which spans roughly 550 kilobases on 19q13 in the order 3, 1, 6, 7, 11, 2, 5, 4, 9 (the precise localization of the two other genes PSG 8 and PSG10 is not yet known). To address the question of PSG regulation, we performed a Clustal alignment of 3 kilobases upstream of the first codons of PSG 1, 2, 3, 4, 5, 6, 7, 9, and 10. The clustering of the promoters indicated a very high level of conservation and was not able to group *PSG4*, *PSG5 *and *PSG7 *as more similar together (not shown). This indicates that the specifically high expression level of 3 genes, which are not contiguous, depends on specific long-range acting chromatin factors.

*ANGPTL4*, also called *FIAF *(fasting-induced adipose factor) is another 3 h hypoxia-activated gene recurrently found in PE cases. It is a downstream target of PPARγ (peroxisome-proliferator-activated receptor γ), and is therefore supposed to regulate lipid metabolism and glucose homeostasis [37]. Although belonging to a family of 9 genes, it is the only one that we could detect by hybridization, suggesting a highly specific mode of regulation, consistently with what has been described of its specific regulation compared to *ANGPTL3 *[38]. In mice, *ANGPTL4*/*FIAF *is increased in the plasma by fasting and decreased by high fat diet [39], demonstrating its involvement in lipid capture in difficult physiological conditions, of which PE may represent a paradigmatic case.

Another unexpected actor of the onset of PE could be *H19*. This RNA-encoding gene is of ill-defined function. It is located in the 11p15.5 cluster of imprinted genes and expressed by the maternal allele, in apparent opposition with *IGFII*. Both genes appear regulated by a common DMR (differentially methylated region), and have opposite effects on fetal growth in mice [20,40]. Both *IGFII *and *H19 *were found in the set of hypoxia-induced genes, consistently with the existence of HIF1α binding sites in their promoters suggesting the ability to respond almost instantaneously to variations in oxygen concentration. For *IGFII*, this is also consistent with its described although controversed angiogenic properties [41,42]. However, while *IGFII *was not PE-specific (and was found expressed in term placentas, pathologic placentas (either PE or vascular IUGR) as well as in early placentas exposed to hypoxia), *H19 *was specifically expressed in PE and strongly in severe PE.

In our study, we could observe that true vascular IUGR results in a drastic reduction of the transcriptional activity of the placenta. In these cases, only mRNAs for *IGFII*, *PSG4*, *PSG5 *and *PSG7*, and mitochondrial genes were present, and no polyA could be detected. Therefore, we could conclude that in vascular IUGR, only a minimal set of genes was transcriptionnally maintained in the placental tissue in order to prevent spontaneous abortion, (i) transcripts from genes of the respiratory cascade, (ii) *IGFII*, one isoform of which is the essential growth factor in placenta [20], and (iii) three genes of the PSG cluster. The absence of polyA in the cDNAs from purely vascular IUGR suggests that the remaining clones are maintained by stabilization of the transcripts rather than at the transcriptional level. This may be substantiated by the observation of *IMP3*cDNA in the transcripts specifically expressed in less severe cases of IUGR. *IMP3 *encodes a factor interacting with the *IGFII *mRNA, possibly stabilizing the transcript, and belonging to an imprinted region on chromosome 7.

In non-purely vascular IUGR, *FTH1 *(ferritin) was expressed at a high level, suggesting the existence of genetic adaptative mechanism to a restricted supply of nutrients. However, ferritin was found twice in the library, without correlation with the oxygen status, showing that this gene does not participate directly in the rapid response to low oxygen concentrations. Ferritin is a major factor for ensuring a sufficient iron store to the neonate at birth. This echoes to a study showing that children presenting a low iron store at birth had low serum ferritin concentrations at 9 months [43], suggesting a risk of iron deficiency in the second postnatal year.

Interestingly, in both pathologies, we found the induction of *FEM1-C*. This gene, discovered as a homologous of a *Caeborhabditis elegans *gene contains KH domains, themselves highly present inside FGIF, the principal inducer of the foetal globin. Again, its presence in the library was not correlated with short hypoxia (3 h). However, we observed that its mRNA concentration was lowered at 48 h hypoxia. Its occurrence in pathological situations could refer to a specific adaptative mechanism aiming at increasing the oxygen capture for the foetus. Its induction fits well with the observed increase in ferritin mRNA, both genes *FEM1-C *and *FTH1*, aiming at building the proteic and prosthetic part of the globin polypeptide, respectively.

Some particularly appealing genes for the diseases studied were present in the membranes but were not revealed by hybridization with specific pathologic samples, as they appear down-regulated in pathological conditions. Among those, we found two clones corresponding to *SERPINE2 *and one to *SERPING1*. These two genes are inhibitors of Serine-Proteases that may play a role in thrombus clearance, and therefore are necessary for adequate circulatory functions. *SERPING1 *(also known as C1-inhibitor) is particularly interesting, since several mutations of this gene are involved in the development of hereditary angioedema (MIM&606860, [44-47]). Oedemas frequently accompanies the preeclamptic condition, therefore specific malfunctions of *SERPINE2 *and *SERPING1 *could represent risk factors for placental diseases. *SERPINE2 *also appeared in our previous study [18] as a gene present in the hypoxic library at the highest number of occurrences. Therefore, it suggests that a gene induced by short-term hypoxia, may be on the contrary down-regulated by chronic hypoxia, such as observed in PE. Recently, we have shown that the same type of regulation is also true for *SERPING1 *(F. Quetin and S. T Chelbi, unpublished results).

We performed a systematic analysis of the 5' regions of the PE and IUGR induced genes. In many of them, we found CpG islands that may be modulators of gene expression. In a future work we shall analyze several of these regions by bisulphite analysis of CpG methylation in normal and pathological cases, to try to give a molecular basis to the observed differences in gene expression. In most cases, we discovered more than one HIF binding site in the various promoters identified. Experimental verification will be needed to evaluate the significance of these binding sites.

A surprising result in our study was the demonstration that genes expressed at a level sufficiently high to be detectable in one at least of our hybridization experiments, are mapped to specific chromosome regions. This is particularly the case for clusters of imprinted genes. In these respects, the involvement of the 11p15.5 cluster in the physiopathology of PE fits well with the recent observation that the invalidation of *p57/kip2*, another gene of this cluster results in "preeclampsia-like" symptoms in mice [48].

In recent studies, several groups have demonstrated that the human genome is organized in large clusters of highly expressed genes [49,50]. This high level organization of the human genome is conserved in other mammalian species, such as cattle and pigs [51]. In the present study, we demonstrate that beside expression level, clustering also exists for functional purposes, such as placental physiology. This vision of the mammalian genome is consistent with the hypotheses and experimental demonstrations developed by Cremer and co-workers, indicating that the presence of entire chromosomes or chromosome regions inside sub-compartments of the nucleoplasm triggers variations in expression levels [52].

## Conclusion

In conclusion, our study has:

• Demonstrated highly significant differences between isolated IUGR and PE concerning the effects of placental oxygen pressure on gene expression. This finding suggests that very different mechanisms are involved when IUGR originates from a fetus-borne developmental dysfunction, and when IUGR results from a vascular defect such as PE.

• Provided the scientific community with a directly available tool, making the link between hypoxia and placental diseases

• Confirmed the importance of several risk factors for PE and IUGR (such as CGA, PSGs and hPL)

• Suggested new possible targets for diagnosing early these pathologies, and possibly for alleviating their effects (such as *COX1*, *ANGPTL4*, *H19*, *FTH1*, *FEM1c*, *IMP3*, *SERPING1 *and *SERPINE2*). Since the SSH was carried out from an early placenta, our study makes the link between potential markers of early oxygen depletion, and the late development of preeclamptic lesions, strengthening the idea that PE may be caused by early alterations of placental function.

• Demonstrated the existence of a genomic organization of placental function in placental mammals.

This work opens the way to characterize at the molecular level the physio-pathological mechanisms of very complex situations that often perturb normal pregnancies.

## Methods

### Patients and ethics

All the placentas from the patients were collected from four Parisian maternities (Cochin, St Antoine, Institut de Puériculture and St Vincent de Paul). This study was approved by the Ethics Committee of Paris Cochin (France), CCPPRB (Comité Consultatif de Protection des Personnes dans la Recherche Biomédicale). All the patients have given their written consent for the use of their placenta. Early term placentas (ETP) were obtained from healthy women undergoing legal abortion by vacuum curettage between 8 and 12 weeks of amenorrhea. "Late" placentas were obtained from caesarean section outside labor from healthy mothers ("FTP or Full-Term Placenta", between 38 and 39 weeks of amenorrhea) or from mothers with pathological pregnancies ("PE" or "IUGR", between 28 and 37 weeks of amenorrhea). The details about the patients used are given on Table 1.

### Placental villi from term placentas (control and pathological)

Biopsy samples were rapidly collected at six to ten various locations from each placenta between the decidual and chorionic plates. Villous tissue was dissected free of fetal membranes, vessels and tissue from maternal origin, rinsed and minced in Ca^2+^, Mg^2+ ^free Hank's Balanced Salt Solution (HBSS).

### Placental villi from early placentas and hypoxia conditions

Floating villi isolated by fine mechanical dissection were cleaned in order to remove fetal membranes, large vessels and tissue from maternal origin. They were rinsed and minced in Ca^2+^, Mg^2+ ^free Hank's Balanced Salt Solution (HBSS). They were plated on 60 mm diameter dishes (0.4 g villi/dish) in 3 ml of RPMI 1640 medium supplemented with 10% (V/V) fetal bovine serum, 25 mM HEPES, 2 mM glutamine and antibiotics (100 IU/ml penicillin and 100 μg/ml streptomycin). They were made hypoxic by placing them in a Lwoff chamber at 37°C and exposed to an oxygen-depleted atmosphere (2% O_2_, 5% CO_2_, 93% N_2_) or maintained at 37°C in normal conditions in humidified 5%CO_2_-95% air during 3 or 48 h. The hypoxia was controlled by checking the atmospheric oxygen pressure at the end of the experiment using an ABL725 gas analyzer (Radiometer, Copenhagen) as previously described extensively [18]).

After incubation or alternatively, directly after dissection, villi from either early or term placentas were dry-frozen in Trizol™ reagent (Life Technologies, Cergy, France) for RNA isolation, and store at -80°C until processed.

### RNA isolation, Poly-A+ preparation, cDNA synthesis and Subtraction experiment

For the SSH experiment [53], the starting material was constituted by villi from a 11 week normal placenta. After dissection, 2 batches of 2 g of villi from different zones of the placenta were cultured for 3 h either in hypoxic or normoxic conditions. Total RNA was extracted from villous tissue using Trizol™ reagent according to the method of Chomczynski and Sacchi [54]. PolyA+ RNAs were then fractionated from total RNA on Oligo dT latex beads using Macherey-Nagel columns (Macherey-Nagel, Germany). cDNA were synthesized from 1.5 – 2 μg of polyA+ using the reverse transcriptase of the cDNA-select kit (PCR-Select cDNA Subtraction Kit, CLONTECH). Linkers were ligated to one batch of each sample ("tester") and hybridized with an excess of the other sample ("driver") using two consecutive hybridization steps. The products obtained are then amplified by PCR using two nested primers present in the linkers. After the SSH procedure, two effects are expected to happen on the cDNAs: firstly, a normalization that reduces drastically the number of molecules corresponding to highly abundant mRNA species, and secondly, a subtraction which enriches considerably each "tester" in tester-specific molecules, i.e. molecules that were initially rare in the "driver". The experiment was then performed by following accurately the manufacturer's advices, and performing all the possible controls at each step. The normalization was monitored by measuring the *GAPDH *level by quantitative RT-PCR in the SSH product versus a mock-subtracted sample. In our case, we could show that the normoxia specific product (called "N-H") was roughly depleted 4,000 times in *GAPDH*, while the hypoxia-specific product (called "H-N") was depleted around 3,000 times in *GAPDH*. The quality of the subtraction was evaluated by RT-PCR using primers for the Vascular Endothelial Growth Factor (*VEGF*) (sense: 5'-ATGAACTTTCTGCTGTCTTGGGTG-3' and antisense: 5'-CTCACCGCCTCGGCTTGTCAC-3'), this gene being highly induced in hypoxic condition. While a band was clearly detectable after 25 PCR cycles in the hypoxia versus normoxia SSH product, no signal could be seen after 40 PCR cycles in the normoxia versus hypoxia SSH product (not shown).

### Library construction and spotting on high-density macroarrays

The secondary PCR product (nested PCR at the end of each subtraction) was cloned in pGEMT vector (Promega corp.), by incubating 300 ng of each of the PCR products with 50 ng of vector with T4 DNA ligase (Biolabs) and the appropriate buffer during 48 h at 4°C, in a total volume of 20 μl. The ligations were diluted to the fifth in sterile water and used to transform DH10B electrocompetent *E. coli *bacteria using a Biolabs electroporator. The transformation titre was evaluated on 8 cm LB-agar plates prepared with 100 μg/ml ampicillin and IPTG/XGal [55], and the next day ~1500 colonies from each of the two subtractions were plated on 22 cm × 22 cm LB-agar trays. After an overnight growth, the colonies were manually picked (864 from the N-H library and 1440 from the H-N library) and grown individually in 96-well mega plates containing 1 ml LB/100 μg/ml ampicillin and 10% glycerol. The plates were covered with porous covers and grown overnight at 37°C under rocking at 200 rpm. Part of the culture was stored at -80°C and 200 μl were transferred to Genetix™ 96-well plates and used by a robotic station for double-spotting on nylon membranes (Hybond N+, Amersham) overlaying new LB-agar/ampicillin trays. The cultures were grown overnight on the membranes at 37°C and the DNA was prepared *in situ *by three consecutive incubations in NaOH 0.5 M (20 min.), Tris-Cl 1.5 M (10 min.), pH 7.5 and SSC 2X (10 min.). Then, the DNA was fixed to the membranes by UV-light irradiation during 5 min (304 nm).

### Probes, Probe labelling and Hybridizations

Early placentas around 11 weeks (2 hybridizations), early placentas exposed to an hypoxic environment during 48 hours (2 hybridizations), or term placentas from normal (3 hybridizations, out of which one correspond to a mixture of 6 term cDNAs) and pathological states placenta (10 PE, 2 PE+IUGR, 3 IUGR, one of which was a mixture of 2 IUGR were hybridized to two sets of membranes. As the clones were spotted in duplicates, each hybridization resulted in four positive signals for an expressed gene. Probes were synthesized from 4 μg of total RNA by random hexanucleotide priming, using 20 μM of primers in the presence of Moloney Murine Leukemia Virus Reverse Transcriptase (MMLV) in a total volume of 25 μl at 39°C according to the manufacturer's specifications (Life Technologies). The cDNA products were stored at -20°C until required for probe labelling. Labelling was performed with 50 ng of cDNA and 5 μl α^33^P dATP using the Biolabs Klenow labelling kit, and allowing the reaction to last during four hours, following the manufacturer's recommendations for ^33^P labelled nucleotides. The ratio of radiolabeled nucleotide incorporation was evaluated by counting before and after TCA precipitation, and was systematically close to 80% [55]. Hybridizations were performed overnight with a standardized amount of radiolabelled probe, in a hybridization oven at 42°C in the following buffer: SSC 4X, Denhardt's 2.5X, SDS, 0.5%, Tris HCL-EDTA (10 mM-1 mM, pH 7.5), Dextran sulfate 1 g/10 ml. The membranes were then washed three times at 58°C in 2X SSC, wrapped in Saran™ and autoradiographed overnight.

### Sequencing

Addresses of positive clones were identified from the autoradiography and the corresponding colonies were grown overnight in 3 ml LB/ampicillin. After overnight growth at 37°C, with a 200 rpm circular agitation, the plasmids were miniprepped according to classical protocols. The cDNA concentration was evaluated after running on an Ethidium Bromide-stained 1% agarose gel, and 600 ng were sequenced using a 16-capillar Applied Biosystem sequencing machine, using an Applied Biosystem sequencing kit.

### Quantification of the signals and statistical analysis

Each signal was quantified by densitometry using the Scion software [56]. Data were entered in an Excel table encompassing 2304 rows and one column for each signal obtained in a hybridization experiment. Data were normalized by reference to a maximum signal intensity fixed at 120 (arbitrary units). They were then grouped into six categories corresponding to the averages of the hybridization experiments, ETP (Early Term Placenta) ETP48 h (Early Term Placenta maintained 48 h at 2% O2), FTP (Control Full Term Placenta), PE (Preeclampsia), R (Intra-Uterine Growth retardation), PER (Preeclampsia and Intra Uterine Growth Retardation). Data were then clustered in a hierarchical tree using the software developed by Eisen and coworkers, Cluster and Treeview, available at [22,57]. The program Cluster was used after the following data preparation: median centering and normalization of genes and arrays, before launching the "complete clustering" procedure. The distribution of groups of genes expressed in only one situation, was analyzed in relation to expected distribution in the normoxic/hypoxic plates of clones (plates 1 to 9 resulted from the normoxia – hypoxia subtraction, while plates 10 to 24 resulted from hypoxia – normoxia subtraction. The distributions could therefore be tested by a Chi2 analysis.

A simple mathematical method was implemented for profiling the CpG islands present in the promoters of the genes identified. Briefly, all the CpG positions were identified in the 5 kb upstream of transcription initiation start site for each gene, and the distance between two consecutive CpG was computed. Then, a mobile average was computed for sliding windows corresponding to ten consecutive CpG islands. A CpG density was estimated at each position by dividing 1000 by the average of each sliding window, resulting in the number of CpG per kilobases of DNA.

To study statistically the chromosomal distribution of the highly expressed subset of placental genes from the macroarrays, we evaluated the size of the minimal statistically significant interval expected to contain 2, 3, 4, 5, 6, 7 or 8 genes. This was calculated using a Binomial law with 2 to 8 successes amongst 276 trials, with a probability of success being estimated as the size of the interval divided by the genome size (3000 megabases; in this study, the gene distribution was supposed to be similar for each chromosome, which is an approximation since some chromosomes are gene-rich and some others are gene-poor). The probabilities were corrected by the Bonferroni correction, available at [58] for multiple testing, assuming 276 independent tests. Using this procedure, the minimal significant genomic interval sizes could be estimated at 0.21, 1.18, 3.05, 5.70, 9.05, 13.0 and 17.30 Mb for 2, 3, 4, 5, 6, 7, or 8 genes respectively (The complete set of data is available upon request).

## Authors' contributions

DV coordinated the program and constructed the SSH libraries, carried out several hybridizations, read the films, analyzed the results at the statistical and biological level and wrote the draft of the article. FM and AGD purify the placental villi from the decidue, put them in culture, purify the total and polyA+ RNA, participated in the SSH and performed a large part of the hybridization experiments. TMM read the films independently and managed the medical files of the patients. BR prepared part of the cDNA from the mRNA samples, as well as HJ. RR took care of the cultures in hypoxia enabling to prepare the cDNA from early villi. STC and FQ contributed to the fine characterization of the membranes. GM and VS helped in the characterization of the membranes by the gift of cDNA samples from JEG-3 cell cultures (results not included in the present paper). FP was in charge of the robotic spotting enabling to construct the membranes. JLD carried out the primary hypoxic culture that were used for the SSH experiments, VR and BC are both clinicians that were in charge of collecting the patients at the Institut de Puériculture and St Antoine hospital, respectively. FF helped in the redaction of the paper.
